# Home Time After an Emergency Department Visit by People Living with Dementia

**DOI:** 10.1002/alz70858_099407

**Published:** 2025-12-25

**Authors:** Justine Seidenfeld, Lindsay Zepel, Valerie Smith, Anita Vashi, Fernanda Bellolio, Courtney H Van Houtven, Susan Nicki Hastings

**Affiliations:** ^1^ Durham VA Healthcare System, Durham, NC, USA; ^2^ Duke University School of Medicine, Durham, NC, USA; ^3^ VA Palo Alto Healthcare System, Palo Alto, CA, USA; ^4^ Mayo Clinic, Rochester, MN, USA

## Abstract

**Background:**

People living with dementia (PLWD) face short‐term risks following emergency department (ED) visits. However, the long‐term impacts of ED visits on PLWD quality of life are not well characterized. Home time‐ days alive and outside hospitals or other care settings‐ is an emerging measure that can reflect longer‐term outcomes. We aimed to identify patient and ED visit characteristics associated with variation in home time after an ED visit.

**Method:**

Retrospective observational study of Veteran PLWD with ≥1 VA ED visit between 10/01/2016 and 09/30/2018. We used VA and CMS data sources to define veteran characteristics and outcomes. Primary outcome was home time in the 180 days after an ED visit, defined as days alive and not in an ED, inpatient, post‐acute, or a nursing home setting. We used a negative binomial model to evaluate the associations between covariates and home time with VAMC‐level random effects to account for correlation among patients at the same VAMC.

**Result:**

Our final cohort comprised 51,707 Veteran PLWD, predominantly male (97.6%) and white (73.0%), with mean age 79.9 (SD 8.59). Most lived in urban areas (74.4%), and 52.2% were married. Characteristics associated with reduced home time included older age (RR 1.06, 95% CI 1.05‐1.07), never married (RR 1.24; 1.16‐1.32) or divorced/widowed (RR 1.24; 1.20, 1.28), depression (RR 1.13; 1.09, 1.17), and ED visits for chronic conditions (RR 1.13; 1.08, 1.18). Female sex (RR 0.88; 0.79‐0.97) and lower clinical frailty (RR 0.79; 0.75, 0.83) were associated with more home time.

**Conclusion:**

Patient and ED visit characteristics impact home time up to 180 days after an ED visit. Older, frailer, unmarried, male patients and those presenting with chronic conditions experienced reduced home time, suggesting directions for targeted interventions. Efforts such as enhanced discharge planning and addressing psychosocial factors (e.g., depression) could improve quality of life.

